# Assessment of Photodynamic Inactivation against Periodontal Bacteria Mediated by a Chitosan Hydrogel in a 3D Gingival Model

**DOI:** 10.3390/ijms17111821

**Published:** 2016-11-01

**Authors:** Po-Chun Peng, Chien-Ming Hsieh, Chueh-Pin Chen, Tsuimin Tsai, Chin-Tin Chen

**Affiliations:** 1 Department of Biochemical Science and Technology, National Taiwan University, Taipei 10617, Taiwan; swaigod@hotmail.com (P.-C.P.); d00b22004@ntu.edu.tw (C.-P.C.); 2 Department of Cosmetic Science, Providence University, Taichung City 43301, Taiwan; chienming.hsieh@gmail.com; 3 Graduate Institute of Biomedical Materials and Tissue Engineering, College of Oral Medicine, Taipei Medical University, Taipei 11031, Taiwan; tmtsai00@gmail.com

**Keywords:** chitosan hydrogel, photodynamic inactivation, periodontal disease

## Abstract

Chitosan hydrogels containing hydroxypropyl methylcellulose (HPMC) and toluidine blue O were prepared and assessed for their mucoadhesive property and antimicrobial efficacy of photodynamic inactivation (PDI). Increased HPMC content in the hydrogels resulted in increased mucoadhesiveness. Furthermore, we developed a simple In Vitro 3D gingival model resembling the oral periodontal pocket to culture the biofilms of *Staphylococcus aureus* (*S. aureus*), *Aggregatibacter actinomycetemcomitans* (*A. actinomycetemcomitans*), and *Porphyromonas gingivalis* (*P. gingivalis*). The PDI efficacy of chitosan hydrogel was examined against periodontal biofilms cultured in this 3D gingival model. We found that the PDI effectiveness was limited due to leaving some of the innermost bacteria alive at the non-illuminated site. Using this 3D gingival model, we further optimized PDI procedures with various adjustments of light energy and irradiation sites. The PDI efficacy of the chitosan hydrogel against periodontal biofilms can significantly improve via four sides of irradiation. In conclusion, this study not only showed the clinical applicability of this chitosan hydrogel but also the importance of the light irradiation pattern in performing PDI for periodontal disease.

## 1. Introduction

Periodontal disease or periodontitis (PD) is an inflammatory disease of the gingival tissue induced by bacteria residing in the plaque biofilm on the subgingival tooth surface. Bacterial infection induces the pocket formation between gingiva and tooth that causes gingival margin retraction and provides an ideal environment for the growth of periodontal bacteria, finally resulting in tooth loss [1,2]. The current treatment of periodontitis is through either the mechanical cleaning of the teeth with systemic antibiotics or a localized delivery system incorporating an antibiotic [1,2]. These conventional treatments for periodontal disease fail to remove periodontal infection in a subset of cases, such as those with complicated root morphology. Meanwhile, the use of antibiotics raises a number of issues, like bacterial resistance to administered antibiotic and unpleasant or toxic side effects. To achieve the therapeutic concentration in the gingival crevicular fluid of the periodontal pockets, large doses of antibiotics will be taken. However, this might cause unwanted side effects and possible antibiotic resistance. In addition, it is not easy to have an effective dose for the topical agents used to remove the biofilm [3,4,5]. These difficulties demonstrate that there was a strong need to develop alternative techniques to mitigate these problems.

Photodynamic therapy combines a photosensitizer (PS) and visible light, which has been developed for various purposes such as antimicrobial disinfection [6,7], anticancer therapy [8], tissue welding, and tissue engineering approaches [9]. Antimicrobial photodynamic inactivation (PDI) as a bactericide employs the nontoxic PS and light irradiation to generate cytotoxic species, which have toxic effects that cause microbial cell death [10]. Many organisms, both bacterial and fungal, found within the flora of the oral cavity have demonstrated susceptibility to various photodynamic treatment regimens In Vitro and In Vivo [11,12,13,14]. Due to the direct binding of PS to the cell wall and membranes, PDI causes direct damage to the cells. It has been shown that repeated PDI does not induce resistance in the bacteria against PDI treatment [15,16]. Due to its selectivity and being harmless to the patient’s sense of taste, PDI has emerged as an alternative treatment for periodontal disease [17].

Dental biofilm is a three-dimensional structure of bacterial communities adhered to the tooth surface [18]. The formation of biofilm is a multistep process involving initial surface attachment, cell growth, and microcolony formation, with resultant maturation into a three-dimensional biofilm. Presently, most PDI studies against periodontal bacteria have only employed planktonic cells or biofilms grown in acrylic disks immersed in broth [19]. However, these PDI studies cannot adequately reflect the situations in the oral cavity, particularly the gingival tissue [20]. In this regard, a suitable gingival model bearing oral biofilms will be helpful to obtain more reliable PDI results in modulating photosensitizer and light delivery.

Chitosan is the partially deacetylated derivative of chitin, which is normally found in the exoskeleton of crustaceans and cell walls of fungi. Chitosan is a polycationic biopolymer, consisting of *N*-acetyl-d-glucosamine and β-1,4-linked d-glucosamine [21]. It has received a great deal of attention for medical and pharmaceutical applications owing to its excellent biocompatibility, negligible immunogenicity, and low cytotoxicity [22,23,24]. Additionally, numerous studies have reported its antimicrobial activity, which make it an attractive biopolymer for the development of drug delivery systems in treating infectious diseases [25,26,27]. Previously, we have shown that 30 min incubation time with chitosan following PDI can potentiate the bactericidal efficacy in planktonic cells and biofilms [28]. Furthermore, we developed a chitosan-based hydrogel containing toluidine blue O (TBO), which shows synergetic PDI efficacy after light irradiation [29]. In this study, we assessed the feasibility of this chitosan hydrogel for the treatment of periodontitis. To this end, a 3D gingival model was established by using putty/light-body impression materials to mimic the periodontal pocket. Our studies showed that microbial biofilm grown on this 3D gingival model provides a more accurate experimental platform for examining the efficacy of photosensitizer formulation as well as modulating light delivery in performing PDI against periodontal infections.

## 2. Results

### 2.1. Mucoadhesion Studies of Chitosan Hydrogel

It is important to maintain a therapeutic concentration of PS when performing PDI against periodontal microbia. In a clinical situation, the salivary flow and the capacity of PS to adhere to the oral target tissue will affect the therapeutic efficacy of PDI. Therefore, a suitable delivery system to maintain the PS in the oral cavity will render PDI effective against target organisms. Previously, we have shown that a chitosan-based hydrogel containing TBO could exert synergetic PDI efficacy [29]. Ideally, low hydrogel hardness and compressibility will ensure that minimum work is more advantageous for hydrogel application and administration, while high mucoadhesive strength and adhesiveness will ensure prolonged adhesion of hydrogel in the gingival pocket. In this study, we adjusted the hydroxypropyl methylcellulose (HPMC) concentration to increase the mucoadhesive property of this chitosan hydrogel. As shown in Table 1, the mucoadhesive strength of chitosan hydrogel containing 0.25% (F-1 formulation) and 0.5% (F-2 formulation) HPMC significantly increased from 5.13 ± 0.09 N to 7.23 ± 0.34 N compared with TBO (0.77 ± 0.04 N) and the mixture of TBO and chitosan (1.45 ± 0.07 N). These results demonstrate that the mucoadhesion increased as the HPMC content increased, which was correlated to its adhesiveness from our previous texture analysis [29]. Meanwhile, the injectability of chitosan hydrogel became difficult when the HPMC concentration is higher than 0.5%.

### 2.2. Effect of Formulations and Postincubation Time on Photodynamic Inactivation (PDI) against Staphylococcus aureus (S. aureus) Biofilms in 3D Gingival Model

**Figure 1:**
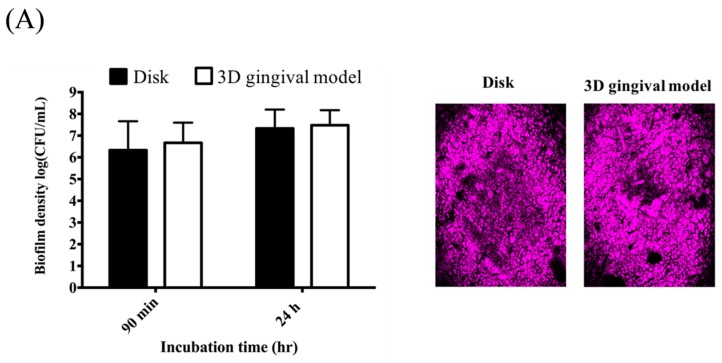

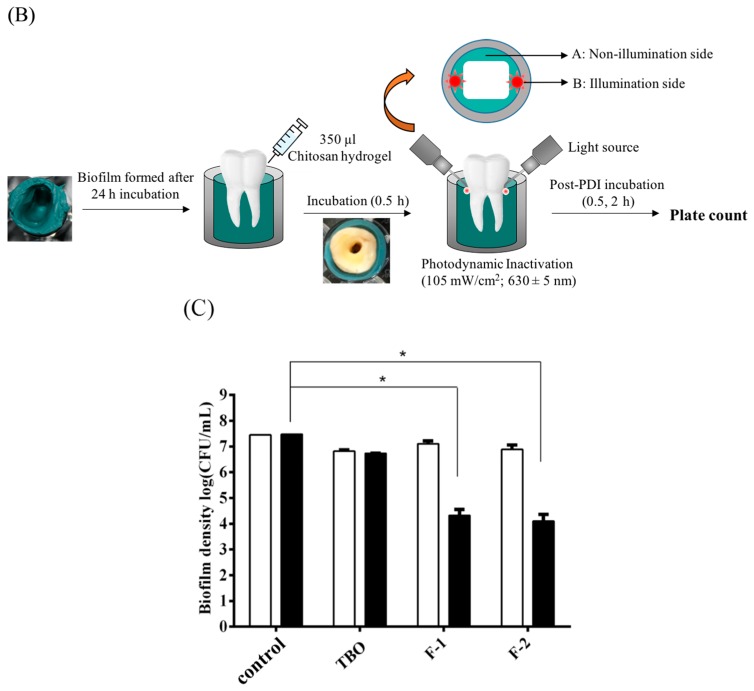
(**A**) Comparison of the biofilm density (**left** panel) and extracellular polymeric substance (**right** panel) of *S. aureus* biofilms cultured in the disk model and 3D gingival model, respectively; (**B**) Schematic illustration of the procedures of chitosan hydrogel mediated photodynamic inactivation (PDI) in 3D gingival model; (**C**) Cell survival fraction of *S. aureus* biofilm in 3D gingival model treated will chitosan hydrogel-mediated PDI. Biofilm cells were incubated with 20 μM TBO or chitosan hydrogels containing 20 μM toluidine blue O (TBO) for 0.5 h, followed by light exposure on two sides of the 3D gingival model (irradiation dose: 5.4 J·cm^−2^ for each side; total light dose: 10.8 J·cm^−2^). After PDI, *S. aureus* biofilm was maintained in contact with the chitosan hydrogels with an additional 0.5 h (☐) and 2 h (■) incubation prior to plate count. Each value is the mean of three independent experiments ± standard deviation. * *p* < 0.05.

A suitable In Vitro model mimicking the periodontal pocket will be helpful to evaluate the PDI efficacy of this chitosan-based hydrogel against periodontal biofilms. In this regard, we used vinyl polysiloxane impression material to develop a 3D gingival model as described in Experimental Section. We first examined the impact of the impressive materials on the growth of *Staphylococcus aureus* (*S. aureus*) biofilms cultured in a 3D gingival model compared with that of cultured in the traditional disk model. Meanwhile, we also examined the extracellular polymeric substance (EPS) of *S. aureus* biofilms cultured in both models using fluorescence microscopy. As shown in Figure 1A, the growth of *S. aureus* biofilms in both models was approximately identical when incubated for 90 min or 24 h. The EPS presence of *S. aureus* biofilms was also found comparable in both models. Accordingly, it clearly demonstrated there was no significant difference between biofilms formed on the disk and the 3D gingival model. The PDI efficacy of chitosan-based hydrogels was then assessed against *S. aureus* biofilms cultured in this 3D gingival model with an additional retention time after PDI, as performed previously [29]. Figure 1B shows the diagram of performing PDI against *S. aureus* biofilm in this 3D gingival model by selected chitosan hydrogel formulations. Approximately 3-log reduction in the survival rate of *S. aureus* biofilm was observed with an additional 2 h incubation using F-1 and F-2 formulation (Figure 1C), while no antimicrobial effect was found with an additional 0.5 h post-PDI incubation. Therefore, the F-2 chitosan hydrogel with a better mucoadhesive property was used for the following studies.

### 2.3. Effect of Illumination Site and Non-Illumination Site on PDI against S. aureus Biofilm

**Figure 2:**
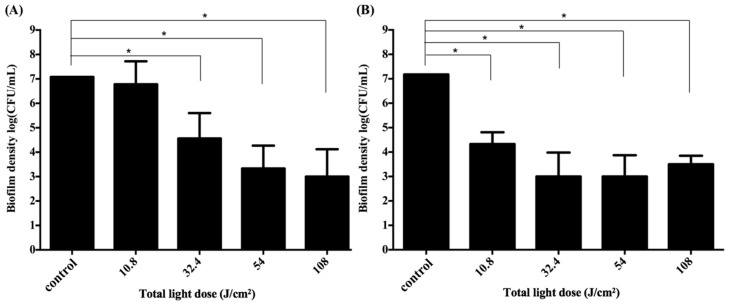
Cell survival fraction of *S. aureus* biofilm in 3D gingival model after incubated with F-2 chitosan hydrogel for 30 min subjected varies dose of light illumination on two sides. After PDI, *S. aureus* biofilm was maintained in F-2 for 0.5 h (**A**) and 2 h (**B**) followed by plate count. Each value is the mean of three independent experiments ± standard deviation. * *p* < 0.05.

In this study, we only found a 3-log reduction against the *S. aureus* biofilm cultured in the 3D gingival model. As PDI efficacy relates to the light dose delivered to the treatment site, we argued whether the insufficient light dose results in the incomplete cell killing in performing PDI at this 3D gingival model. As shown in Figure 2, there was a 2–3-log increase in PDI efficacy when the total light dose increased from 10.8 to 32.4 J·cm^−2^. However, the survival rate of *S. aureus* biofilm did not further reduce at a higher light dose, regardless of whether the post-incubation time was 0.5 h or 2 h, respectively. These results indicate that a higher light dose did not lead to an increase in PDI efficacy as expected.

**Figure 3:**
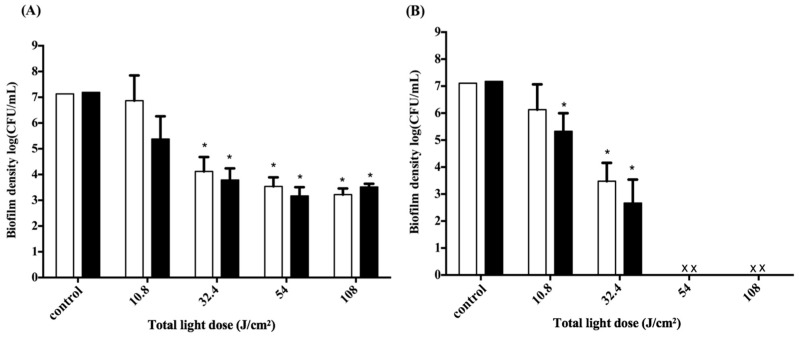
Cell survival fraction of *S. aureus* biofilm in 3D gingival model after incubation with F-2 for 0.5 h, and subjected to various doses of light illumination from two sides. After PDI, *S. aureus* biofilm was maintained in contact with F-2 for 0.5 h (☐) and 2 h (■) prior to plate count. (**A**) Non-illumination side; (**B**) illumination side. The locations of (**A**) and (**B**) are shown in Figure 1B. Each value is the mean of three independent experiments ± standard deviation. * *p* < 0.05.

Previously, we have shown that the complete inactivation of traditional *S. aureus* biofilm was observed with an additional 1 or 2 h incubation when performing PDI with this chitosan hydrogel [29]. We further addressed whether the loss of light-dose-dependence was due to incomplete killing of the bacterial biofilm grown in this 3D gingival model. Therefore, we first compared the cell survival fraction of *S. aureus* biofilms in both the illumination site and the non-illumination site after PDI treatment (Figure 1B). Figure 3 showed the plate counts of *S. aureus* biofilms in non-illumination and illumination sites after PDI. The survival of *S. aureus* biofilms at the non-illumination site decreased gradually as total light dose increased from 10.8 to 32.4 J·cm^−2^ (Figure 3A). The PDI efficacy was about 3-log reduction at the non-illumination site under the total light dose of 32.4 J·cm^−2^ (16.2 J·cm^−2^ per side). However, the survival rate of *S. aureus* biofilm at the non-illumination site still remained 10^3^ colony-forming unit (CFU)/mL even when increasing the total light dose to 108 J·cm^−2^ (54 J·cm^−2^ per side). On the other hand, a significant light-dose-dependent reduction of the *S. aureus* survival rate was found at the illumination site (Figure 3B). A complete eradication could be found when the total light dose increased to 54 J·cm^−2^ (27 J·cm^−2^ per side), suggesting at least 27 J·cm^−2^ of light dose per side might be required to reach a complete killing at the illumination site. These results imply that an incomplete cell-killing at the non-illumination site might be the main reason for the inefficiency of PDI, as found in Figure 2.

### 2.4. Adjustment of PDI Treatment

**Figure 4:**
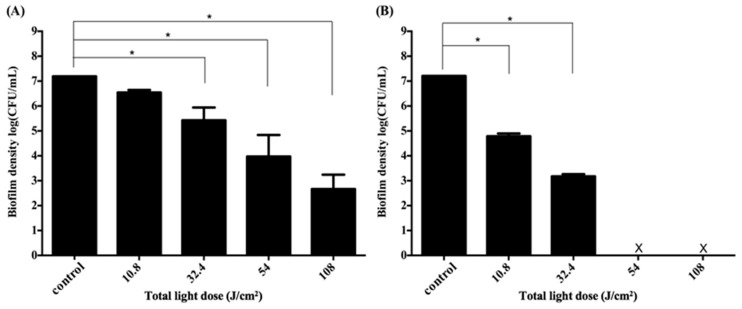
Cell survival fraction of *S. aureus* biofilm cultured in a 3D gingival model after incubation with F-2 formulas for 0.5 h and subjected to various doses of red-light illumination from four sides. After PDI, *S. aureus* biofilm was maintained in F-2 for 0.5 h (**A**) and 2 h (**B**) followed by plate count. Each value is the mean of three independent experiments ± standard deviation. * *p* < 0.05.

Since the difference in cell survival fraction of *S. aureus* biofilm was found between the illuminated and non-illuminated sites in the 3D gingival model, we further adjusted the light irradiation pattern from two to four sites, which allowed illumination of the 3D gingival model equally from four sides. The responses to PDI using F-2 against *S. aureus* biofilm following with 0.5 h and 2 h incubation are shown in Figure 4A,B, respectively. Light-dose-dependent killing of *S. aureus* was found in both treatments; meanwhile, a better antimicrobial effect was found with longer post-incubation after PDI. Clearly, the efficacy of PDI against *S. aureus* biofilm was increased when the total light dose was increased. The PDI efficacy against *S. aureus* biofilms was about 2-log and 4-log reduction by illuminating a total light dose of 32.4 J·cm^−2^ (8.1 J·cm^−2^ per side) with 0.5 h and 2 h post-incubation, respectively. The complete eradication was achieved when a higher total light dose irradiation (>54 J·cm^−2^) was applied; namely, *S. aureus* biofilm can be completely eradicated by illuminating either 16 or 27 J·cm^−2^ of light dose per side with 2 h post-incubation. These results correlate with our previous findings that the augmented PDI efficacy relates to the post-incubation time of chitosan hydrogel and the damage level induced by PDI [23,28,29].

### 2.5. Impact of the Light Dose on PDI against Periodontal Strain Biofilms

**Figure 5:**
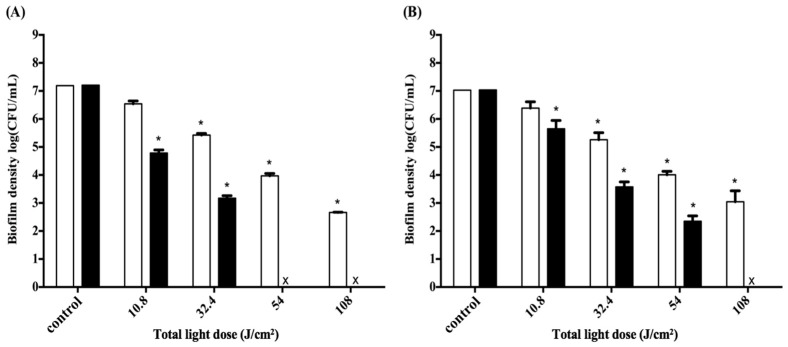
Cell survival fraction of *Aggregatibacter actinomycetemcomitans* (*A. actinomycetemcomitans*) (**A**) and *P. gingivalis* (**B**) biofilms in a 3D gingival model after incubation with F-2 for 0.5 h, and subjected to various doses of light illumination from four sides. After PDI, biofilm maintained contact with F-2 for 0.5 h (☐) and 2 h (■) prior to plate count. Each value is the mean of three independent experiments ± standard deviation. * *p* < 0.05.

To further evaluate the PDI efficacy on the biofilms of periodontal bacterial strains, we used two major periodontopathic pathogens, *Aggregatibacter actinomycetemcomitans* (*A. actinomycetemcomitans*) and *Porphyromonas gingivalis* (*P. gingivalis*), which are Gram-negative oral anaerobes that are involved in the pathogenesis of periodontitis [30,31]. Figure 5 shows the survival rates of *A. actinomycetemcomitans* and *P. gingivalis* biofilms treated with F-2 chitosan hydrogel followed by various light doses with four sides of irradiation. The survival of *A. actinomycetemcomitans* and *P. gingivalis* in biofilms both gradually decreased in a light-dose-dependent manner with 0.5 h of post-incubation with chitosan hydrogel. A total light dose irradiation higher than 32.4 J·cm^−2^ was required to observe a significant drop in survival rate in both periodontal strain biofilms. As for the 2 h of post-incubation with chitosan hydrogel, the results show that viability of both biofilms significantly decreased compared to control at various light doses. Encouragingly, complete eradication was found when the total light dose increased to 54 and 108 J·cm^−2^ for *A. actinomycetemcomitans* and *P. gingivalis* biofilm, respectively.

## 3. Discussion

Antimicrobial PDI has been shown as an alternative modality for treating microbial infections. The main advantages of PDI are that bacteria can be eradicated almost instantly and that damage to adjacent host tissues can be minimized. In the dental field, a great number of studies have shown that oral bacteria are susceptible to PDI when suspended in planktonic cultures [32], oral biofilms [33], as well as carious dentine [34]. Among the photosensitizers, TBO and methylene blue (MB) are the most commonly used for oral antimicrobial PDI [35,36,37]. Nevertheless, several studies have demonstrated incomplete PDI destruction of oral pathogens in biofilms using MB [38,39] and TBO [40,41]. Except for the drug-resistant mechanisms, the EPS amount and thickness of the biofilm can affect the penetration and diffusion of PS throughout the entire biofilm [42]. In present clinical application, TBO and MB were normally prepared as aqueous solution, which might restrict the retention and penetration of PS in oral biofilms as they might not have enough retention time to attach onto the biofilm on the tooth. In addition, the presence of proteins derived from gingival crevicular fluid and saliva could also interfere their penetration [13]. Therefore, it is worthwhile to develop a suitable delivery system to improve the pharmacological characteristics of the PS and overcome the incomplete eradication of dental plaque microorganisms. The adhesive characteristic is an important parameter in designing an oral delivery system, since a desirable contact and retention in the periodontal pocket will ensure better clinical efficacy [43]. Chitosan, a natural derivative of chitin, is a polysaccharide that has been shown to possess a broad spectrum of antimicrobial activity against fungi, yeast, and bacteria [44,45,46]. Costa et al. used chitosan as an alternative to local conventional antimicrobials against oral anaerobic bacteria [47]. In their study, chitosan showed great promise as an alternative natural antimicrobial for anaerobic pathogens, particularly against anaerobic pathogens involved in oral infections. In addition, Sarasam et al. evaluated the antibacterial activity of chitosan-based matrices on oral pathogens, which suggested antibacterial properties of chitosan are contact-dependent and can vary with different species of oral bacteria [48]. As a result, greater antibacterial properties of the porous 3D scaffolds compared to 2D membranes were observed, owing to more surface area for bacterial adhesion. The enhanced mucoadhesive property of chitosan is attributed to increased retention of the formulation, which leads an important position in the arena of oral drug delivery [49]. We have previously showed that longer retention of a chitosan hydrogel containing TBO could increase the antimicrobial PDI effect [29]. In the present study, we further demonstrated that the mucoadhesive ability of this chitosan hydrogel increased with higher HPMC concentration, from 0.25% to 0.5% (Table 1). This result is strongly correlated to the hydrogel bio-adhesiveness when compared to our previous adhesiveness result [29] and the published literature [50]. In addition, the significant antibacterial effect of chitosan was demonstrated when comparied with TBO and control group (Figure 1C). A longer retention of chitosan hydrogel (F-1 and F-2 with 2 h post-incubation) can effectively improve the antimicrobial effect.

Previously, we have shown that an initial 2- to 4-log reduction by PDI was required to result in the complete killing augmented by chitosan [28]. Moreover, the minimum concentration of chitosan might vary, depending on the damage level induced by PDI and bacterial strains used. Meanwhile, we also found that chitosan alone could maximally induce 1-log reduction in planktonic cells without PDI, depending on the microbial strains used [23,28]. In this regard, it is possible that the chitosan hydrogel developed in this study might have some bactericidal effect without PDI. This might explain why the post-illumination effect of chitosan hydrogel seems to be larger in higher doses of illumination, especially against periodontal pathogens.

Under two-sides illumination, a complete cell-killing could be found at the illuminated site when the total light dose was 54 J·cm^−2^ (27 J·cm^−2^ per side) with 0.5 h post-incubation (Figure 3B). However, as shown in Figure 4, with a total light dose of 108 J·cm^−2^ under four-sides illumination, a complete cell-killing could be found with 2 h post-incubation time (Figure 4B) but not with that of 0.5 h (Figure 4A). As mentioned above, the augmented PDI cytotoxicity relates to the chitosan concentration and its post incubation time [23,28]. In this regard, the difference between Figure 3B and Figure 4A with 27 J·cm^−2^ per side at the illumination site suggests that 0.5 h post-incubation time might not be enough to ensure the complete cell-killing with chitosan hydrogel. We have previously shown that the complete inactivation against *S. aureus* biofilm was observed with an additional 1 or 2 h incubation when performing PDI with this chitosan hydrogel [29]. In the future, 1 h post-incubation time might be required to ensure the augmented PDI efficacy induced by chitosan hydrogel.

Presently, most PDI studies against periodontal bacteria have only employed planktonic cells or biofilms grown in acrylic disks immersed in broth [19]. These studies performing PDI—including PS administration and light irradiation—cannot adequately reflect the physiological situation in the oral cavity, particularly the 3D gingival morphology [20]. In this study, we used nontoxic dental impression materials to establish a 3D gingival model to mimic the three-dimensional organization of biofilm adhered to the tooth surface. There is no difference in the bacterial viability of biofilms cultured on gingival model and acrylic disk (Figure 1A). Using this 3D gingival model, we further demonstrated different cell survival rates of the biofilms between the illuminated and non-illuminated sites after PDI (Figure 3). In spite of a higher light dose applied, the bacteria at non-illuminated site were not eradicated completely, leaving some of the innermost bacteria alive. These results implied that the light might be blocked by the tooth in our 3D gingival model, which limited the PDI effectiveness. Different light sources and photosensitizing agents have been used to increase the PDI efficacy for controlling dental biofilm [39]. With this 3D gingival model, we demonstrated that the efficacy of PDI against *S. aureus* biofilm could be enhanced after adjusting light illumination from two-sides to four-sides using the chitosan hydrogel. A complete eradication was also achieved when a higher light dose irradiation (>54 J·cm^−2^) was applied with 2 h of post-incubation. A similar effect was also found when PDI was applied to two major periodontopathic pathogens, *A. actinomycetemcomitans* and *P. gingivalis*. The chitosan hydrogel-mediated PDI was indeed shown to potentiate the PDI efficacy in biofilms of *A. actinomycetemcomitans* and *P. gingivalis* (Figure 5). These results demonstrate that this 3D gingival model not only could be useful to culture periodontal biofilms but also to evaluate the bactericidal efficacy in performing PDI.

Our present results indicate that chitosan hydrogel containing TBO may be a promising delivery system in PDI against periodontal biofilms, particularly when used in clinical practice. In addition, the data of this study clearly demonstrates that adjusting light application onto dental biofilms is crucial for efficient antimicrobial PDI, suggesting appropriate dosage parameters are important in developing PDI for treating periodontal infection in clinical.

## 4. Experimental Section

### 4.1. Preparation of Chitosan Hydrogel and Mucoadhesion Studies

Various hydrogel formulations were prepared using HPMC, chitosan, and TBO as reported previously [29]. HPMC was purchased from F. D. Enterprise Corporation. Chitosan (molecular weight: 25–35 kDa) with degree of deacetylation >90% was obtained from Shin Era Technology (Taipei, Taiwan). TBO and acetic acid were purchased from Sigma-Aldrich (St. Louis, MO, USA). F-1 and F-2 were prepared by mixing 60 μM TBO solution, 0.75% (*w*/*w*) chitosan/acetic acid solution, and desired HPMC solution (volume ratios of HPMC:chitosan:TBO = 1:1:1). The hydrogel mixtures were stirred at room temperature until dissolved completely and then left to swell for at least 24 h at room temperature before further experiments. For comparison, a final concentration of 20 μM of TBO content was prepared throughout the experimental period.

The mucoadhesive strength of the formulations was evaluated by measuring the force required to detach the formulation from a mucin disk using a TAXT2 plus Texture Analyser (Stable Micro Systems Ltd., Godalming, UK) equipped with a 5 kg load cell in tension mode. Texture profile analysis (TPA) may be applied for the mechanical characterization of pharmaceutical gels and a semisolid system [43,51]. Mucin disks were manufactured by compression of mucin (250 mg) using a single punch tablet machine (Carver Inc., Wabash, IN, USA) with a 13 mm die and a compression force of 10 tones, applied for 30 s. The disks were then horizontally attached to the lower end of the cylindrical probe (length 5 cm, diameter 1 cm) using double-sided adhesive tape. The samples, packed into the small cylindrical vessels, were placed under the upper probe. After the mucin disk made contact with the sample for 120 s, the probe was then moved at a constant speed of 0.1 mm/s. The peak value in the force—time plot determined the result. All the above three experiments from the formulations were conducted in triplicates at 37 °C. Table 1 shows the compositions and mucoadhesive properties of each formulation used throughout this study.

### 4.2. Development of the 3D Gingival Model

**Scheme 1:**
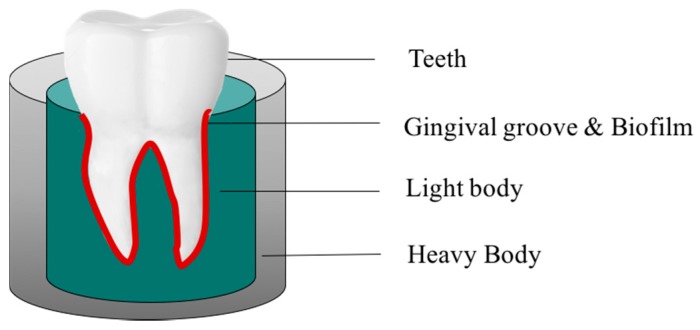
Schematic representation of the biofilm-3D gingival model.

The vinyl polysiloxane impression material (Correct VPS putty), purchased from Pentron Clinical (Orange, CA, USA), was used to develop a 3D gingival model. First, putty base and catalyst were mixed well and uniformly filled into the 48-well multitier plate. Once the putty base was settled, the tooth with wax coating on the surface was then inserted into the base. The 3D gingival model was left to solidify at room temperature and last for 24 h. Finally, the wax was removed by hot water, which formed the space of the gingival groove. Scheme 1 represents the biofilm-3D gingival model employed for the study.

### 4.3. Biofilm Formation and EPS Staining in Biofilms

*S. aureus* (BCRC 10780) was purchased from the Bioresource Collection and Research Center (Hsinchu, Taiwan). *Porphyromonas gingivalis* (*P. gingivals* ATCC 33277) and *A. actinomycetemcomitans* (ATCC 10953) were provided by Dr. Yee-Chun Chen from the NTU Hospital (Taipei, Taiwan). Tryptic soy broth (TSB), purchased from Difco (Detroit, MI, USA), was used as the liquid culture medium for *S. aureus*, *P. gingivals*, and *A. actinomycetemcomitans*.

Bacterial biofilm grown on a traditional disk reactor was modified from Pitts et al. design [52]. The composition of the reactor was a 500 mL polypropylene container, a Teflon rotor, and 24 removable 316 L stainless steel disks inserted on the rotor. For traditional biofilm formation, one milliliter of *S. aureus* seed culture was inoculated into the rotating disk and incubated at room temperature overnight as described previously [29]. Then, the reactor was continuously fed with 0.01× TSB at a rate of 240 mL·h^−1^. After 24 h, the biofilms could reach a steady state and the averaged biofilm density was 5 × 10^7^ CFU/mL. To culture the biofilm in the 3D gingival model, 250 μL bacterial suspension containing 10^8^ CFU/mL was loaded into the gingival groove and incubated 24 h. After incubation, the bacterial suspension was removed and the resident biofilm was gently washed twice with sterile phosphate-buffered saline (PBS) and refilled with 250 μL fresh culture medium. The biofilm density and extracellular polymeric substance in the 3D gingival model and the traditional disk model were compared at 90 min and 24 h, respectively.

EPS in biofilms were examined by a Leica SP2 confocal scanning fluorescence microscope (Leica Inc., Malvern, PA, USA) equipped with a 20× or 40× water-dipping objective lens. The SYPRO^®^ Ruby biofilm matrix stain (Invitrogen Corporation, Grand Island, NY, USA) was used to stain proteins in the EPS for 60 min and excited at 405 nm with diode laser. The stained biofilms were then rinsed in the phosphate buffer for 10 min before observations.

### 4.4. Chitosan Hydrogel-Mediated PDI in Biofilm Cells

Each biofilm was treated with 350 μL of chitosan hydrogel via injection into the gingival groove. After 30 min of incubation in the dark, the biofilm-3D gingival model was then irradiated with a diode laser (105 mW/cm^2^; 630 ± 5 nm). The light sources were delivered either from two sides or four sides of the 3D gingival model. As a consequence, the total irradiation dose was counted as the sum of the irradiation from different positions. After light irradiation, the models continued to incubate for an additional 0.5 h or 2 h in the dark. The biofilms were finally removed by vigorously vortexing with 10 mL sterile PBS in the test tubes. The resulting bacterial suspensions were diluted and plated on TSB agar, and the colonies formed after 18 h of incubation at 37 °C were counted. The number of viable cells was determined by averaging the CFU on three plates as described below.

The CFU counting of a bacterial suspension was similar as reported previously [29]. Briefly, 10 μL aliquots from samples with appropriate dilution rate (from 10^−1^ to 10^−6^) were plated on TSB agar plates and further incubated at 37 °C in an incubator for 18 h. The surviving rate was calculated as N_PDI_/N_0_, where N_0_ is the CFU cm^−2^ in the initial sample and N_PDI_ is the CFU cm^−2^ after PDI. The control group, defined as the intrinsic chitosan hydrogel toxicity, was monitored by evaluating the surviving fraction of bacterial samples without light irradiation and calculated as N_DARK_/N_0_, where N_DARK_ is the CFU cm^−2^ of the non-illuminated samples.

### 4.5. Statistics Analysis

For statistical analysis, the result was obtained from three independent experiments and expressed as the mean ± standard deviation. Differences between two means were assessed for significance by the two-tailed Student *t*-test. A *p* value of <0.05 was considered statistically significant relative to control.

## 5. Conclusions

A chitosan hydrogel as well as a 3D gingival model were developed for evaluating the PDI efficacy against dental biofilms. Optimized PDI procedures employed on the model for periodontal treatment were also investigated under various adjustments of the energy and distribution of light. Overall, this study has shown the clinical PDI benefits of the bioadhesive chitosan hydrogel formulation for the treatment of periodontal disease in this In Vitro gingival model.

## Figures and Tables

**Table 1:** Composition and mucoadhesive characteristics of the chitosan hydrogel and comparison with control groups.

Formulation	HPMC ^a^ (%, *w*/*w*)	Chitosan (%, *w*/*w*)	TBO (μM)	Mucoadhesive Strength ^b^ (N) ±SD
TBO	0	0	20	0.77 ± 0.04
Mixture of TBO and chitosan	0	0.25	20	1.45 ± 0.07
F-1	0.25	0.25	20	5.13 ± 0.09
F-2	0.5	0.25	20	7.23 ± 0.34

^a^: HPMC: Hydroxypropyl methylcellulose; ^b^: Each value is the mean from three independent experiments ± standard deviation; TBO: toluidine blue O.
